# The General Medical Council

**Published:** 1902-12-06

**Authors:** 


### The Hospital.

A JOURNAL OF

The Medical Sciences and Hospital Administration.

VOL. XXXIII.—NO. 845.] EDITED BY SIR HENRY BURDETT, K.C.B., AND SOLOMON C. SMITH, M.D., M.R.C.P. [DECEMBER 6, 1902.

### THE GENERAL MEDICAL COUNCIL.

AT the meeting of the General Medical Council on the 25th instant it became the duty of the President, not, we should think, without some feeling of relief, to announce that the passage of the Midwives Act would render it unnecessary that a question which in time past had occupied so much of the attention of the Council should for the future find any place in its programme of business or on its Minutes. At the same time he described a curiously anomalous position which has been created by the Act, in the provision that the rules framed by the Central Midwives' Board should be submitted, not to the Council as a whole, but to the English Branch Council, before they receive that sanction from the Privy Council which is necessary to their validity. The English Branch Council will therefore be required to meet and consider these rules, and, in the words of the President, it cannot be expected that the members of the Branch will devote time to this purpose without receiving some indemnification, while charges for printing, stationery, postages and clerical assistance will also have to be met. No provision has been made by the Midwives Act for the expenses hence arising; and the members of the branch will either be required to give their services for the indicated purpose, or they must receive payment from the funds of the General Council itself; that is to say, from moneys contributed by the medical profession for totally different purposes, and the yearly claims upon which are now in excess of the yearly income, so that they can only be met by a constantly recurring drain upon the capital provided by the accumulations of the past. Why the medical profession, instead of the public, should be called upon to pay for the examination of the rules laid down by the Midwives' Board, is one of the many questions which it "passes the wit of man" to answer in any intelligible manner; but such appears to be the present state of the law, and to be one of the net results of a great many years of wrangling in and out of Parliament over questions which, to say the least, were very imperfectly understood by most of those who took a leading part in discussing them. For the present, however, a long-desired end has been so far attained that, after the first of next April, provision will be made for the creation of a class of certified midwives, with regard to whose competence and character the public will have some sort of security, and who, by the natural operation of events, may be expected to become more and more capable as time goes on. After April 1st, 1905, it will be penal for any woman to take or use the name or title of midwife, or to state that she is recognised by law as a midwife, unless she is duly certified under the Act; and, from April 1st, 1910, it will be penal for any woman habitually and for gain to attend women in childbirth, otherwise than under the direction of a qualified medical practitioner, unless she is duly certified under the Act. In other words, medical practitioners, especially those whose work extends widely over country districts, will be relieved of an enormous amount of badly-paid responsibility, and will be able, without offence either to law or to public opinion, to delegate a large proportion of duties which now press upon them with great severity. It cannot be expected that the gain will be unmixed, and hence it may in some instances entail a loss of cases which some practitioners might desire to retain.

The other important matter coming under the consideration of the Council arises out of the Medical Act passed this year by the Dominion of Canada, and which has received the Royal assent. The primary object of the Act is to do for the several provinces of the Dominion what was done by the Act of 1858 for the United Kingdom, that is to say, to render the qualifications given in each province available in the rest. The provisions of the British Act appear, *mutatis mutandis*, to have been somewhat closely copied; and a Canadian Medical Council has been constituted, with the duty of so controlling medical education in all parts of the Dominion as to secure an approximate uniformity of education and examination in every province. If this were all, the profession at home, while cordially approving of the end in view, would regard the steps taken to secure it with comparatively languid interest. The Dominion, however, is more ambitious, and looks forward to the recognition of Canadian medical qualifications in this country and in other colonies; a prospect clearly fraught with nothing but advantage, if only the necessary standards can be securely maintained. The "if" in question is a large one, and will be rendered larger as each colony follows the example, in the way of extending privileges of practice to the graduates and licentiates of the United Kingdom, which has already been set by India, Malta, New Zealand, and Australia, and by which they have brought themselves under the provisions of the second part of the Medical Act of 1886. We may justifiably feel complete confidence that the medical authorities of the colonies, speaking generally, would be as solicitous as those at home to preserve a high standard of qualification; but, with the examples of some of those at home before our eyes, we cannot forget that a chain is no stronger than its weakest link, and that individual licensing bodies may sometimes be tempted to undue leniency. It would seem that a real reciprocity of medical qualifications throughout the Empire might be best secured by a central Council possessing the reality instead of the mere semblance of authority. If still distant, the day is nevertheless approaching when the Council of the United Kingdom will be rendered powerless by the exhaustion of its pecuniary resources; and it would seem that the conditions hence likely to arise will render necessary, at no remote period, a complete review of the existing state of the law. We may hope that such an opportunity would be employed for the purpose of rendering medical education throughout the Empire commensurate with the growth of science, and independent of the rivalries of corporations.

